# The Foreign Journals: Medical Statistics

**Published:** 1842-07

**Authors:** 


### MEDICAL STATISTICS.

*Results of Revaccination in the Prussian Army in* 1841.

IN the year 1841, out of 44,941 men, who had been vaccinated, and were revaccinated in that year, 23,383, or 52 in 100, exhibited a genuine pock, which went duly through all its stages. The results of this year, therefore, confirm those of the several preceding ones, that the number of cases requiring *re*-vaccination is gradually on the increase. Thus in 1840, there were 48 in 100; in 1839, 46; in 1838 and 1837, 45; in 1836, 43; in 1835, 39; in 1834, 37; in 1833, 31.

*Medicinische Zeitung.* No. xix. *Mai* 11, 1842.

